# Genetic association between celiac disease and chronic kidney disease: a two-sample Mendelian randomization study

**DOI:** 10.1080/0886022X.2024.2357246

**Published:** 2024-06-04

**Authors:** Zhimin Chen, Zigui Zheng, Bingjing Jiang, Yanfang Xu

**Affiliations:** a Department of Nephrology, Blood Purification Research Center, the First Affiliated Hospital, Fujian Medical University, Fuzhou, China; b Research Center for Metabolic Chronic Kidney Disease, the First Affiliated Hospital, Fujian Medical University, Fuzhou, China; c Department of Nephrology, National Regional Medical Center, Binhai Campus of the First Affiliated Hospital, Fujian Medical University, Fuzhou, China

**Keywords:** Celiac disease, chronic kidney disease, estimated glomerular filtration rate, Mendelian randomization, causal relationship

## Abstract

**Objective:**

A two-sample Mendelian randomization (MR) analysis was performed to elucidate the causal impact of celiac disease on the risk of chronic kidney disease (CKD).

**Methods:**

The study comprised data from three genome-wide association studies involving individuals of European ancestry. The study groups included participants with celiac disease (*n* = 24,269), CKD (*n* = 117,165), and estimated glomerular filtration rate levels based on serum creatinine (eGFRcrea, *n* = 133,413). We employed four widely recognized causal inference algorithms: MR-Egger, inverse variance weighted (IVW), weighted median, and weighted mode. To address potential issues related to pleiotropy and overall effects, MR-Egger regression and the MR-PRESSO global test were performed. Heterogeneity was assessed using Cochran’s Q test.

**Results:**

We identified 14 genetic variants with genome-wide significance. The MR analysis provided consistent evidence across the various methodologies, supporting a causal relationship between celiac disease and an elevated risk of CKD (odds ratio (OR)_IVW_ = 1.027, *p* = 0.025; OR_weighted median_ = 1.028, *P* = 0.049; OR_weighted mode_ = 1.030, *p* = 0.044). Furthermore, we observed a causal link between celiac disease and a decreased eGFRcrea (OR_IVW_ = 0.997, *P* = 2.94E-06; OR_weighted median_ = 0.996, *P* = 1.68E-05; OR_weighted mode_ = 0.996, *P* = 3.11E-04; OR_MR Egger_ = 0.996, *P* = 5.00E-03). We found no significant evidence of horizontal pleiotropy, heterogeneity, or bias based on MR-Egger regression, MR-PRESSO, and Cochran’s Q test.

**Conclusion:**

The results of this study indicate a causal relationship between celiac disease and an increased risk of CKD.

## Introduction

Celiac disease, also known as celiac sprue or gluten intolerance, is a chronic intestinal disorder primarily characterized by an immune response to gluten protein [1]. In individuals with celiac disease, upon consumption of foods containing gluten, the immune system initiates an abnormal reaction, resulting in inflammation and damage to the mucosa of the small intestine. This immune-mediated inflammatory response further affects the structure and function of intestinal epithelial cells, resulting in the impaired absorption of nutrients [2]. Clinical manifestations often include diarrhea, abdominal pain, weight loss, and malnutrition, which can significantly compromise the quality of life of affected individuals. However, as research into celiac disease deepens, it has become increasingly evident that the effects are not solely limited to the small intestine [3]. In recent years, a growing body of evidence has suggested that celiac disease may have remote influences on other organ systems, with particular attention being focused on its potential relationship with kidney health.

The kidneys are vital excretory organs within the human body; they play a pivotal role not only in filtering waste and excess substances from the bloodstream but also in maintaining critical functions such as electrolyte balance, water equilibrium, and acid–base balance [4]. Chronic kidney disease (CKD) is a prevalent and severe health concern affecting millions of people worldwide. Epidemiological data has revealed that the prevalence of CKD varies from 7.2% to 13.4% across different countries. In numerous nations, there is a shortage of kidney replacement services, resulting in an estimate of 2.3 to 7.1 million premature deaths among adults unable to access such treatments; therefore, CKD poses a significant burden on global public health management [5, 6]. Currently, the Kidney Disease: Improving Global Outcomes (KDIGO) organization defines CKD as either a sustained decrease in the estimated glomerular filtration rate level based on serum creatinine (eGFRcrea) <60 [mL/min]/1.73 m^2^ for >3 months, or the presence of markers of kidney damage, such as proteinuria, for >3 months, with urinary protein excretion exceeding 30 mg/24 h, or a eGFRcrea persistently <60 [mL/min]/1.73 m^2^, even if lasting <3 months. eGFRcrea is estimated using the four-variable Modification of Diet in Renal Disease Study Equation [7, 8].

Not only does CKD significantly compromise quality of life, it also increases the risk of cardiovascular diseases, skeletal abnormalities, and mortality. CKD can arise from various diseases and factors, including diabetes, hypertension, glomerulonephritis, and polycystic kidney disease. These underlying causes contribute to ongoing structural and functional damage to the kidneys, ultimately impairing the organ’s ability to efficiently eliminate waste and fluids, leading to a spectrum of clinical symptoms and complications [9].

In recent years, several observational studies have suggested a potential link between celiac disease and kidney health [10–13]. Patients with celiac disease are at an increased risk of developing kidney disease, particularly IgA nephropathy; this risk depends on the phenotype of celiac disease. Understanding the potential renal manifestations should be considered when treating patients with celiac disease [12]. Individuals with celiac disease may face an increased risk of kidney damage, which is potentially due to abnormal immune system activation leading to inflammatory responses that affect the structure and function of renal glomeruli. Additionally, patients with celiac disease often experience a chronic state of inflammation, which could have long-term effects on the kidneys [14].

Despite some existing research supporting the association between celiac disease and kidney impairment, investigations in this area remain relatively limited and lack high-quality evidence, such as Mendelian randomization (MR) studies to definitively establish this relationship. MR is rooted in the fundamental principles of Mendelian genetics, where genetic variations are randomly distributed in the transmission of genetic information, analogous to randomization in natural experiments [15]. MR employs a natural experimental design based on genetic variation to assess causal relationships and test causal hypotheses. By leveraging the random distribution of genetic variation, MR simulates the characteristics of a randomized controlled trial, allowing for a reduction of confounding factors and the avoidance of reverse causation. The current understanding of celiac disease suggests that a strong genetic association is with specific variations in the *HLA* genes, such as *HLA-DQ2* and *HLA-DQ8* [16, 17]. These variations are implicated in the immune response to gluten proteins in wheat, leading to the onset of celiac disease. Similarly, the genetic features of CKD involve multiple genes and genetic variations, including *APOL1* and *ACTN4*, contributing to the genetic susceptibility to CKD. MR based on genetic associations is less susceptible to common biases, such as selection bias and information bias, in observational data, enhancing the credibility of the findings [18–20].

The primary objective of this study was to elucidate the causal relationship between celiac disease and kidney impairment, thereby bridging the gaps in our current understanding. We intend to employ a rigorous analytical approach, accounting for potential confounding factors, and assess the robustness of our findings through sensitivity analyses. Through this innovative research design, we aimed to minimize the influence of confounding variables, facilitating a more accurate assessment of the impact of celiac disease on kidney impairment.

## Methods

### Data source

CKD is a progressive decline in kidney function, with a reduction in the eGFR commonly regarded as a key indicator of CKD progression [6, 8]. To further clarify and strengthen the causal relationship between celiac disease and CKD, the present study employed MR inference by utilizing eGFRcrea as an independent outcome factor in relation to celiac disease. We opted to analyze datasets sourced from different populations of the same ethnic group to mitigate selection bias and enhance the generalizability of our findings, thereby rendering them applicable to a broader spectrum of populations or contexts. The genetic association of celiac disease stems from a large-scale genome-wide association study (GWAS) involving 24,269 individuals of European ancestry (Table 1). The GWAS data were derived from the 1000 Genomes Project, and through the utilization of dense resequencing data for fine-mapping GWAS regions, 13 novel celiac disease risk loci of genome-wide significance were identified, bringing the total number of known loci to 40. Celiac disease was diagnosed based on standard clinical, serological, and histopathological criteria, including small intestinal biopsy [21, 22]. Similarly, GWAS data for CKD phenotypes [23] (12,385 cases and 104,780 controls) and eGFR levels [23] (133,413 individuals) were obtained as outcome variables from a European ancestry population (Table 1). The diagnostic criterion for CKD is eGFRcrea <60 [mL/min]/1.73 m^2^. Summary data for all three phenotypes were extracted from the corresponding original studies.

**Table 1.** Detailed data information of the Mendelian randomization study.

Phenotype	Sample size	Sample Case	Sample Control	Ancestry	Number of GVs	Reference
Celiac disease	24269	12041	12228	European	38037	[21]
Chronic kidney disease	117165	12385	104780	European	2179497	[23]
Glomerular filtration rate	133,413	–	–	European	2116469	[23]

### Instrumental variables (IVs) for celiac disease

**Figure 1.**
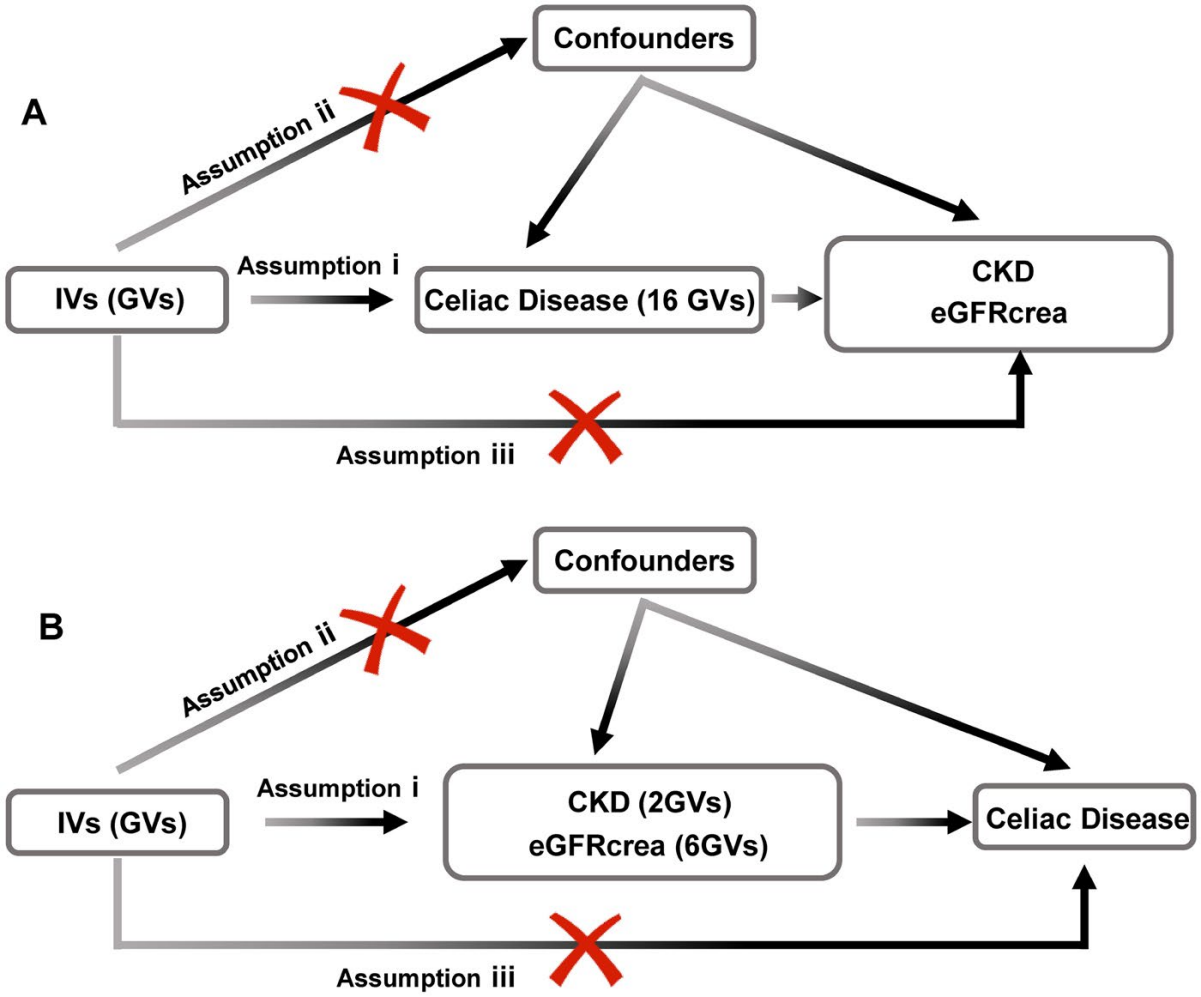
Flowchart of the bidirectional two-sample Mendelian randomization study. The design hypotheses are that the genetic variants are associated with celiac disease traits, but not with confounders, and the genetic variants are associated with the risk of CKD and the eGFRcrea only through celiac disease traits. Similar hypotheses are applicable for the reverse MR analysis. Assumption i: IVs are significantly associated with celiac disease at a genome-wide level; assumption ii: IVs must remain independent of any confounders; assumption iii: IVs solely influence CKD and eGFRcrea through celiac disease. GV, genetic variants; CKD, chronic kidney disease; eGFRcrea, estimated glomerular filtration rate levels based on serum creatinine; IV, instrumental variables.

To employ genetic variation for assessing the causal relationship between the exposure (celiac disease) and outcomes (CKD, eGFRcrea), three pivotal assumptions must be met for the IVs [20]: (i) IVs are significantly associated with celiac disease at a genome-wide level; (ii) IVs must remain independent of any confounders; and (iii) IVs solely influence CKD and eGFRcrea through celiac disease (Figure 1). Reverse MR follows the same rules. Due to the presence of linkage disequilibrium structures within the genome, notable associations between genetic variations and traits at the threshold of *P* = 5 × 10^−8^ and r^2^ < 0.001 were found to exist. Subsequently, utilizing the R software package TwoSampleMR, we identified single nucleotide polymorphism loci related to celiac disease occurrence, satisfying the first assumption.

### MR analysis

The statistical analysis employed a two-sample MR approach to investigate the genetic relationship between celiac disease and the risk of CKD, in addition to eGFRcrea. The primary analytical method was the inverse variance weighted (IVW) method. The IVW estimation aggregates the ratio estimates of each genetic variant into an overall estimate using an IVW formula, where the weight of each genetic variant is the reciprocal of its variance of the ratio estimate [24]; this aids in analysis precision. It is the default method in TwoSampleMR and the most commonly used for MR. While including multiple variants in the MR analysis increases the statistical power, it introduces the potential for pleiotropy, i.e., where a single genetic variant is associated with more than one variable. To explore and adjust for pleiotropy, we employed complementary analytical methods, such as MR-Egger, weighted median, and weighted mode. MR-Egger regression analysis provides robustness against invalid instruments and tests by estimating a summary data-based causal effect through a weighted linear regression of gene outcome coefficients on gene exposure coefficients. The slope of this regression represents the causal effect estimate, while the intercept can be interpreted as an estimate of the average pleiotropic effect across genetic variations. In an ideal scenario, if there is no pleiotropy, this slope should approximate zero. If the slope significantly deviates from zero, it indicates the presence of horizontal pleiotropy, wherein one or more genetic variants (GVs) exhibit associations with outcomes beyond the direct relationship between exposure and outcome. The weighted median estimator offers consistent estimates of causal effects, even when up to 50% of the analytical information stems from invalid IVs. Compared to the MR-Egger analysis, the weighted median estimator retains higher precision in estimation. A significance level of *P* < 0.05 is considered statistically significant for testing.

### Sensitivity analysis

Subsequently, we conducted sensitivity analyses to indirectly test the second and third assumptions. Firstly, we employed the MR-PRESSO global test and MR-Egger regression to examine the presence of horizontal pleiotropy. MR-PRESSO is a method utilized to detect potential exogenous bias in MR analyses; it identifies outliers in the genotype–phenotype association, which are data points that deviate from the expected relationship with other variables. By correcting for these outlier data points, MR-PRESSO enhances the accuracy and robustness of causal inference [25]. On the other hand, MR-Egger is employed to address potential pleiotropy within genetic IVs. By providing a robust method for causal estimation, MR-Egger can effectively operate even in the presence of some degree of pleiotropic bias within the IVs [26]. The simultaneous use of these two sensitivity analysis methods allows for a comprehensive assessment of the robustness of the MR analysis results, thereby mitigating potential bias effects and enhancing the credibility of causal inference. Secondly, Cochran’s Q test was employed to assess heterogeneity among IVs. Furthermore, a ‘leave-one-out’ analysis was performed to investigate the possibility that the causal relationship is driven by a single GV. A significance level of *p* < 0.05 was considered indicative of significant heterogeneity and pleiotropy.

### Reverse MR

The methodology of the data analysis was similar to the aforementioned approach, with the distinction being that the GWAS data for CKD and eGFRcrea were utilized as exposure factors, while celiac disease served as a singular outcome event. This was employed to assess the reverse causal relationship of CKD and eGFRcrea with the risk of celiac disease.

### Ethical approval

Our analysis utilized publicly available GWAS summary data. Each GWAS included in this study had obtained relevant ethical approval from the respective institutional review boards in the original studies, and all participants provided informed consent.

## Results

### IVs of MR

We selected 16 independent GVs from the GWAS of celiac disease as IVs; two GVs were subsequently excluded during the merging process with the outcome event. In a genome-wide context, all these genes are associated with celiac disease (Table 2). The causal effects of each GV on CKD and eGFRcrea are depicted in the forest plot. Generally, an F-statistic <10 for each individual variable was used to define ‘weak IVs’, and the use of weak IVs in a study can lead to substantial bias [27]. In our study, after screening, the 14 selected GVs all had F-values >30, rendering weak instrument bias negligible.

**Table 2.** Summary of genetic variants used to instrument celiac disease.

GV	CHR	POS	EA	OA	EAF	BETA	SE	*P*-value	F statistic
rs10752747	1	2524915	T	G	0.309	−0.116	0.020	5.05E-09	34.176
rs10892258	11	118579865	A	G	0.233	−0.150	0.022	1.73E-11	45.253
rs11979905	7	37437877	G	A	0.108	0.163	0.029	2.49E-08	31.070
rs12527282	6	137967252	T	C	0.280	−0.155	0.021	1.69E-13	54.346
rs12663317	6	32742827	C	A	0.085	−0.616	0.043	3.53E-47	208.225
rs130078	6	31118565	G	C	0.781	−0.577	0.024	3.35E-125	566.441
rs13030124	2	204694263	A	G	0.450	−0.105	0.019	2.40E-08	31.136
rs13119723	4	123218313	G	A	0.153	−0.309	0.028	7.60E-29	124.100
rs13198474	6	25874423	A	G	0.054	0.946	0.031	1.00E-200	920.516
rs2441467	2	61374149	C	T	0.418	0.105	0.019	1.70E-08	31.801
rs6498114	16	10964118	T	G	0.756	−0.131	0.021	5.83E-10	38.378
rs7162232	15	75115895	A	G	0.681	−0.117	0.020	7.97E-09	33.282
rs9296009	6	32114515	T	A	0.234	−0.954	0.029	1.00E-200	1087.020
rs931	6	33054619	A	G	0.307	0.618	0.020	1.00E-200	979.696

### MR results

**Figure 2.**
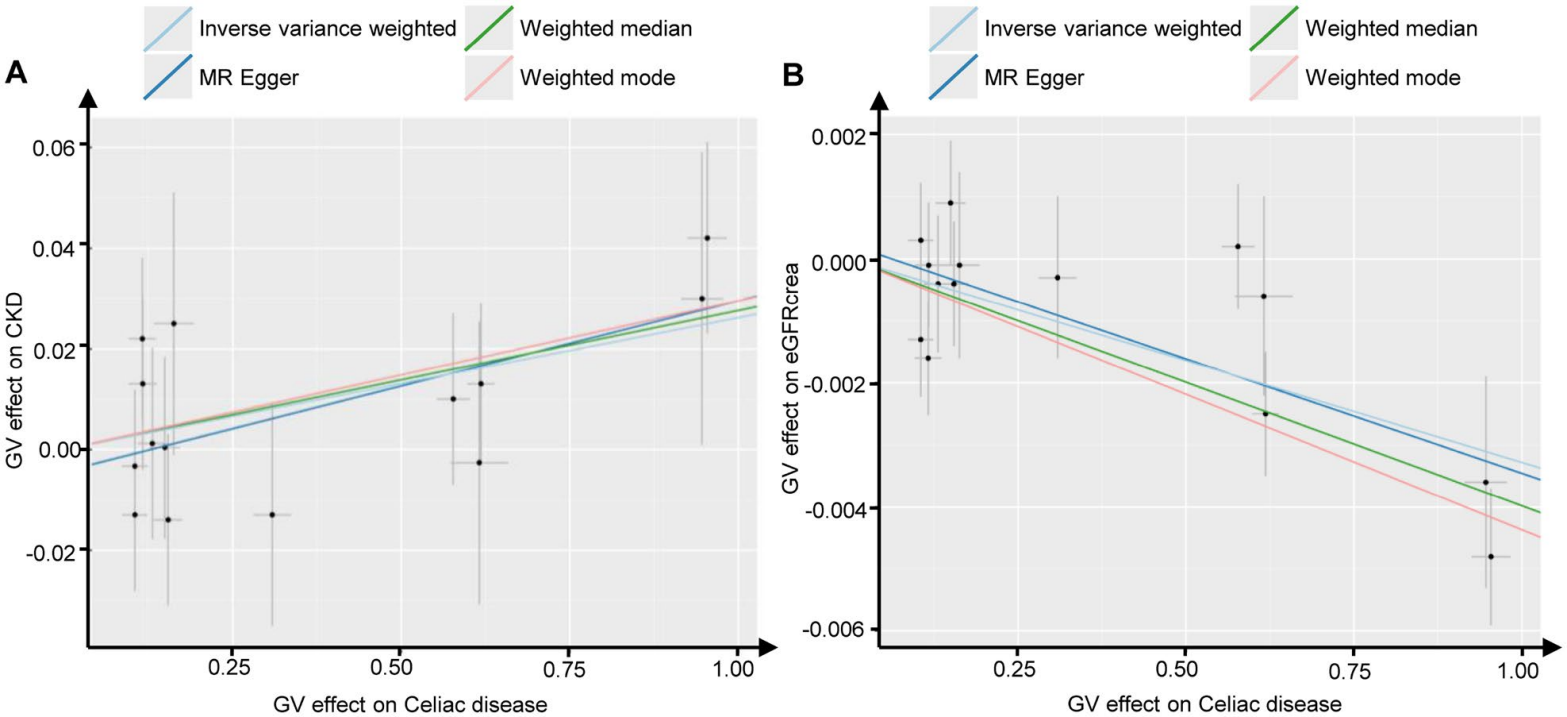
Scatter plots of Mendelian randomization. Scatter plot estimations of celiac disease as the expositional factor on CKD (A) and eGFRcrea (B). Each point in the scatter plot represents an IV. The line on each point reflects the 95% CI. A positive slope indicates that celiac disease had a positive effect on CKD. A negative slope indicates that celiac disease has a detrimental impact on eGFRcrea levels. GV, genetic variants; CKD, chronic kidney disease; eGFRcrea, estimated glomerular filtration rate levels based on serum creatinine; IV, instrumental variables; CI, confidence interval.

Among the four algorithms, evidence from the three distinct algorithms, excluding MR-Egger (odds ratio (OR)_MR-Egger_ = 1.034, 95% confidence interval (CI): 1.000–1.070, *P* = 0.075), supported a causal relationship between celiac disease and an increased risk of CKD (OR_IVW_ = 1.027, 95% CI: 1.003–1.050, *P* = 0.025; OR_weighted median_ = 1.028, 95% CI: 1.000–1.057, *P* = 0.049; OR_weighted mode_ = 1.030, 95% CI: 1.004–1.057, *P* = 0.044). The scatter plot revealed a positive correlation between the risk of CKD occurrence and the celiac disease phenotype. Celiac disease was also found to be causally associated with a reduced eGFRcrea (OR_IVW_ = 0.997, 95% CI: 0.995–0.998, *P* = 2.94E-06; OR_weighted median_ = 0.996, 95% CI: 0.994–0.998, *P* = 1.68E-05; OR_weighted mode_ = 0.996, 95% CI: 0.994–0.997, *P* = 3.11E-04; OR_MR-Egger_ = 0.996, 95% CI: 0.994–0.998, *P* = 5.00E-03) (Table 3, Figure 2).

**Table 3.** Causal effects of bidirectional Mendelian randomization.

Exposure	Methods	nGVs	OR	95%CI	*P*-value
CD versus CKD	MR Egger	14	1.034	0.999-1.070	0.07514414
	Weighted median	14	1.028	1.000-1.057	0.04958771
	Inverse variance weighted	14	1.027	1.003-1.050	0.02469948
	Weighted mode	14	1.030	1.003-1.057	0.04401706
CD versus eGFRcrea	MR Egger	14	0.996	0.994-0.998	5.00E-03
	Weighted median	14	0.996	0.994-0.998	1.68E-05
	Inverse variance weighted	14	0.997	0.995-0.998	2.94E-06
	Weighted mode	14	0.996	0.994-0.997	3.11E-04

### Heterogeneity and pleiotropy analysis

**Figure 3.**
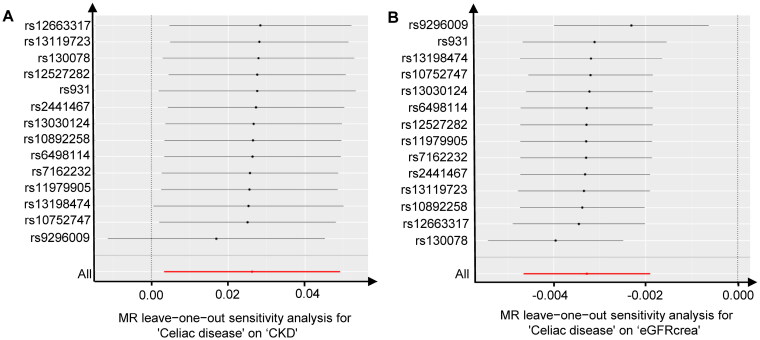
Leave-one-out tests of the genetic risks of celiac disease on CKD and eGFRcrea. The red lines are the analysis results of random effects IVW. CKD, chronic kidney disease; eGFRcrea, estimated glomerular filtration rate levels based on serum creatinine; IVW, inverse variance weighted.

Subsequently, we conducted sensitivity analyses to assess the robustness of our findings. Cochran’s Q test indicated no evidence of heterogeneity among the individual variant IV estimates (celiac disease to CKD: *P*_IVW_ = 0.894, *P*_MR-Egger_ = 0.870; celiac disease to eGFRcrea: *P*_IVW_ = 0.408, *P*_MR-Egger_ = 0.352). The intercept represents the average pleiotropic effect of genetic variation (the average direct effect of the variation associated with the outcome). A non-zero intercept (MR-Egger test) indicates directional pleiotropy. The MR-Egger regression demonstrated that directional pleiotropy was unlikely to have introduced bias into the results (celiac disease to CKD: intercept = −4.36E-03; *P* = 0.561; celiac disease to eGFRcrea: intercept = 2.31E-04; *P* = 0.620). Furthermore, the MR-PRESSO global test results indicated the absence of overall pleiotropy across all instrumental variables (celiac disease to CKD: Rssobs = 8.477, *P* = 0.889; celiac disease to eGFRcrea: Rssobs = 18.949, *P* = 0.315). This outcome suggests that intravenous injections are unlikely to influence the CKD risk and eGFRcrea levels through pathways other than celiac disease. The results of the ‘leave-one-out’ analysis indicated that there was no single GV driving the IVW point estimate (Table 4, Figure 3).

**Table 4.** Pleiotropy and heterogeneity tests of Mendelian randomization.

Exposure	IVW_Q_pval	MR Egger_Q_pval	egger_intercept	egger_intercept _pval	RSSobs	Global *P* value
CD versus CKD	0.894	0.870	−4.36E-03	0.561	8.477	0.889
CD versus eGFRcrea	0.408	0.352	0.000	0.619	18.949	0.315

### Reverse MR

When utilizing CKD as the exposure factor, only two significant IVs were identified; however, MR analysis with celiac disease as the outcome event failed to reveal a causal relationship between them (OR_IVW_ = 0.978, 95% CI: 0.708–1.352, *P* = 0.895). Similarly, employing eGFRcrea as the exposure factor led to the identification of six significant IVs, yet MR analysis with celiac disease as the outcome event also failed to ascertain a causal association between them (OR_IVW_ = 1.097, 95% CI: 0.225–5.350, *P* = 0.909; OR_weighted median_ = 1.961, 95% CI: 0.240–16.051, *P* = 0.530; OR_weighted mode_ = 3.875, 95% CI: 0.202–74.465, *P* = 0.410; OR_MR-Egger_ = 8.572, 95% CI: 0.072–1021.247, *P* = 0.428).

## Discussion

Through the MR design employed in this study, we have deeply assessed the causal relationship between celiac disease and CKD, shedding light on potential interactions between the two diseases. Within the genetic markers of celiac disease, we meticulously selected an independent set of GVs as IVs, ensuring a randomized distribution to mitigate the influence of confounding factors. Supported by various algorithms and sensitivity analyses, we have arrived at a series of pivotal conclusions.

Firstly, we have observed a causal relationship between celiac disease and the risk of CKD. While the significance of the MR-Egger method may not be prominent, the concurrence of results from the IVW, weighted median, and weighted mode methods underscore the reliability of this causal association. Given the precision advantages preserved in estimations through methods such as the IVW method, the consensus among these three approaches is robust. Furthermore, we have established a causal linkage between celiac disease and a reduction in the eGFRcrea. The alignment of results across multiple algorithms further deepens our comprehension of the potential impact of celiac disease on kidney function. This suggests a plausible role of celiac disease in the decrement of the eGFR, thereby strengthening the proposition of its latent influence on kidney health. Despite individual methods in the MR analysis using CKD as the outcome showing *P*-values close to 0.05, our MR analysis using eGFRcrea as another independent indicator yielded significant results. This confirmed a causal relationship between celiac disease and a lower eGFRcrea, thus validating the robustness of our findings. Additionally, the reverse MR analysis, employing CKD and eGFRcrea as singular exposure factors, further reinforces a unidirectional causal relationship between celiac disease and kidney health. In summary, our study harnesses the power of MR to elucidate the causal dynamics between celiac disease and CKD. The amalgamation of diverse analytical methods and rigorous sensitivity analyses bolsters the credibility of our findings, contributing to a more comprehensive understanding of the intricate interplay between celiac disease and kidney health [28].

From a mechanistic standpoint, celiac disease is an autoimmune disorder marked by inflammation in the mucosa of the small intestine, which stems from an abnormal immune response to gluten proteins [11]. Unlike inflammatory bowel disease, where MR studies suggest no causal relationship with CKD [29], the intestinal inflammation caused by celiac disease arises from abnormal immune system activation, which renders the kidneys particularly sensitive to such immune dysregulation. Inflammatory mediators, such as tumor necrosis factor-alpha (TNF-α) and interleukin-1 (IL-1), may enter the bloodstream and affect renal tissues, inducing kidney inflammation and damage. Prolonged inflammatory states can foster fibrosis and glomerulosclerosis, ultimately resulting in a gradual decline in renal function [30]. Furthermore, dysregulation of the immune system in individuals with celiac disease may trigger the deposition of immune complexes within the kidneys. These complexes can initiate glomerular inflammation and localized immune reactions, causing disruption and impairment of the glomerular filtration barrier. Glomerular filtration barrier dysfunction can give rise to symptoms such as proteinuria and hematuria, consequently elevating the risk of chronic kidney failure.

Celiac disease may also prompt changes in the composition of the gut microbiota, affecting the balance of microbial communities [31]. Imbalanced microbiota can contribute to increased intestinal permeability, allowing harmful substances, such as endotoxins, to enter the bloodstream. This can lead to systemic inflammatory responses, exerting adverse effects on the kidneys. Furthermore, individuals with celiac disease might experience insulin resistance and hyperglycemia, which are closely associated with the development of diabetic kidney disease [32]. Hyperglycemia can potentially damage the renal tubules and increase the permeability of the glomerular filtration membrane, thereby impacting normal kidney function. Additionally, disruptions in glucose metabolism may exacerbate renal inflammation and oxidative stress, contributing to impairment of the renal tissue [33]. In summary, the elevated risk of chronic kidney failure in individuals with celiac disease may arise from the intricate interplay of various mechanisms. These mechanisms may be interconnected, collectively influencing the structure and function of the kidneys [34].

Interestingly, our reverse MR results demonstrate that CKD does not lead to celiac disease. This may be attributed to the impaired excretion of waste and fluids in CKD, resulting in electrolyte disturbances and metabolic acidosis, but not directly affecting the digestive system’s processing of food. In contrast, celiac disease is an autoimmune disorder primarily caused by the small intestine’s hypersensitivity to gluten, leading to impaired nutrient absorption and digestive issues. In addition, the kidneys are highly susceptible to damage in the presence of autoimmune abnormalities, such as those caused by anti-neutrophil cytoplasmic antibody or systemic lupus erythematosus, further exacerbating renal impairment [35, 36]. Additionally, it is worth noting that the reverse MR analysis did not identify additional GVs, which may be associated with weak instrument bias, and batch effects between datasets from different sources may also contribute to less accurate results. Nonetheless, for a comprehensive understanding of the relationship between celiac disease and CKD, further extensive research is essential to validate these mechanisms and investigate more specific biological processes [37].

While this study has yielded significant findings through the utilization of MR design and a variety of analytical methods, it is imperative to recognize the presence of certain limitations that could potentially impact the interpretation and generalization of the conclusions. Although MR design has the capability to mitigate the influence of confounding factors, it falls short in providing direct evidence of causal relationships. However, MR experiments are typically conducted over a relatively short time frame, thus potentially limiting their ability to capture long-term treatment effects and disease progression. Additionally, patients with celiac disease or CKD may present with concurrent health issues, such as hypertension or diabetes, which could confound study outcomes [38]. Nevertheless, these factors are challenging to mitigate through MR experiments. In this context, the study relies on statistical methodologies to infer the potential association between celiac disease and CKD. To mitigate these limitations, we have employed sensitivity analyses, such as the MR-Egger method, to assess and adjust for potential pleiotropy. Additionally, we have conducted power calculations to ensure adequate statistical power for our analyses and have carefully selected instruments with strong genetic associations to minimize the risk of weak instrument bias. Therefore, further clinical investigations are necessary to validate these inferred associations. Furthermore, in exploring the putative mechanisms underlying the heightened risk of CKD attributable to celiac disease, this study posits certain hypotheses; however, the specific biological mechanisms remain incompletely elucidated [39]. While we have advanced conceivable mechanisms, their validation demands more extensive experimental and molecular investigations. Furthermore, the study’s sample predominantly comprises specific populations, which could potentially limit the external validity of the research findings. Genetic backgrounds, lifestyles, and environmental factors across divergent populations may potentially engender distinct impacts on the relationship between celiac disease and CKD. In light of these considerations, future research endeavors should aim to corroborate these findings more comprehensively and contemplate strategies to mitigate these limitations. This is crucial for facilitating a more nuanced comprehension of the intricate interplay between celiac disease and CKD. In summary, while this study contributes valuable insights, its conclusions are tempered by certain constraints. Rigorous validation efforts and strategies to address these limitations are imperative for a more comprehensive understanding of the intricate relationship between celiac disease and CKD.

## Conclusion

In the present study, the application of MR analysis unveiled a potential association between celiac disease and an increased risk of CKD. This revelation introduces a novel perspective that contributes to a deeper understanding of the interplay between the gastrointestinal and renal systems, offering potential guidance for therapeutic strategies in individuals with celiac disease. Nonetheless, further investigation into the underlying mechanisms through experimental validation is imperative to enhance our comprehension of this intricate relationship.

## Supplementary Material

Supplemental Material

## Data Availability

The data underlying this article were accessed from the IEU OpenGWAS consortium (https://gwas.mrcieu.ac.uk/).
